# Prognostic implications of negative dobutamine stress echocardiography in African Americans compared to Caucasians

**DOI:** 10.1186/1476-7120-6-20

**Published:** 2008-05-20

**Authors:** Ajay V Srivastava, Karthik Ananthasubramaniam, Salil J Patel, Natesh Lingam, Gordon Jacobsen

**Affiliations:** 1 Heart and Vascular Institute, Henry Ford Hospital, Detroit, USA; 2 Department of Biostatistics & Research Epidemiology, Henry Ford Hospital, Detroit, USA

## Abstract

**Background:**

African Americans (AA) have higher rates of cardiovascular morbidity and mortality than Caucasians (CA). Despite its excellent negative predictive value, the influence of race on the prognostic implications of negative dobutamine echocardiography in predicting major cardiac problems is largely unknown.

**Methods:**

We studied 387 AA and 340 CA patients with negative dobutamine stress echocardiography (NDSE). Kaplan-Meier survival analysis was used to create freedom-from-event curves for major adverse cardiac events over a 36-month period, and a Cox proportional-hazards multivariable model to examine the influence of race on cardiac outcomes.

**Results:**

AA patients were younger (69.4 ± 12.6 vs. 74.2 ± 10.7, p < .001), had higher incidence of diabetes mellitus (37% vs. 29%, p = .01), hypertension (91% vs. 85%, p = .006), left ventricular hypertrophy (70% vs. 49%, p < .001) and lower incidence of prior coronary artery disease (27% vs. 34%, p = .05) compared to CA patients. Ejection fraction ≥ 50% was comparable (81% vs. 82%, p = .8). At 3-years, AA patients had a lower freedom from nonfatal myocardial infarction (92% vs. 96%, *p *= .006) and any cardiac event (cardiac death, myocardial infarction) (91% vs. 95%, *p *= .005) compared to CA patients.

**Conclusion:**

This is the first study to demonstrate that AA patients have higher rates of nonfatal MI and MACE compared to CA patients with a NDSE. These patients require closer follow-up and aggressive preventive and treatment strategies should be employed to help reduce cardiovascular morbidity and mortality despite negative ischemic workup.

## Background

Cardiovascular disease accounts for significant morbidity and mortality in the United States [1,2] and is a major cause of death in all race groups [3,4]. Prior studies have shown that racial differences exist among the various ethnic groups and African Americans (AA) have the highest cardiac event rates [5-7]. Overall mortality in AA's has been attributed to racial disparities and other differences [5-12]. AA patients have a higher incidence of diabetes mellitus, hypertension, and obesity [13,14], all of which are risk factors for cardiovascular mortality, and prior reports have shown that reduced access to health care and lower socio-economic status [13] also contribute to worse outcomes in this group.

Though there is data demonstrating that AA patients have a lower incidence of obstructive coronary artery disease (CAD) compared to Caucasian (CA) patients [15], they still have significant rates of myocardial infarction and other cardiac events. This has been partially attributed to a difference in the pathology of coronary arteriosclerosis in AA's compared to CA's. Prior studies have shown that AA's have a higher prevalence of traditional cardiovascular risk factors [13,14], that collectively play a role causing dysfunction at the microvasculature level, and thereby contribute to endothelial dysfunction. These factors concurrently with an unstable plaque may play a role in higher events rather than the continuing progression of coronary artery plaque build-up as seen in CA's [16]. This dissimilarity in pathology between the 2 groups could have potential implications when interpreting diagnostic stress tests performed to rule out CAD.

Stress testing primarily aims to identify significant stenosis causing blunting of stress-induced flow reserve; myocardial perfusion imaging detects CAD based on regional heterogeneities in tracer uptake based on flow reserve abnormalities and stress echocardiography depends on ischemic wall motion abnormalities to develop. It has been demonstrated that when compared to CA's, AA's have lesser significant epicardial CAD in the presence of abnormal nuclear perfusion scans indicating that other factors such as endothelial dysfunction may play a role in these abnormal functional studies [17].

DSE (Dobutamine stress echocardiography) is routinely used for risk stratification [17,18] carrying an approximate cardiac event rate of 1.1% to 1.5% per patient/year, a mortality rate of 0.13% per patient/year. A NDSE is associated with a high negative predictive value [19-21], thereby adding valuable prognostic information. Yet, there is no reported literature to our knowledge comparing major adverse cardiovascular events during follow-up between AA and CA patients with a NDSE. Given the above-mentioned differences in cardiovascular disease process and testing between the two ethnic groups, it would be crucial to examine its implications on the prognostic value of a NDSE.

## Methods

### Study Population

This study was carried out at a large tertiary care center involving an unselected patient population. Between January 1, 1999, and December 31, 1999, 1000 consecutive patients who underwent DSE were screened retrospectively. After applying exclusion criteria (Patients with a positive DSE, younger than 18 years old, pregnant, mentally impaired) 756 patients formed the initial study group. Patients who underwent very early revascularization (< 2 months) after the index-NDSE were also excluded as these are driven by multiple factors including patient presentation, high clinical suspicion in the setting of a NDSE. Given very early revascularization following a NDSE they were excluded to ascertain the natural long-term outcomes following NDSE. Only the first event data was used for patients with more than one event. After excluding patients of other ethnicities, 727 patients with NDSE formed the final study group. Indications for performing the DSE included chest pain (36.2%), pre-operative clearance (17.3%), evaluation for shortness of breath (6.8%), evaluation for CAD (21.9%), and miscellaneous causes (17.8%). The institutional review board at the Henry Ford Hospital approved the study. For purposes of our study, hypertension was defined as office visit documentation of hypertension history or patient being on antihypertensive therapy. Diabetes mellitus was assumed to be present if there was documentation of diabetes mellitus in an office note, or the patient was on antihyperglycemics (oral anti-diabetics or insulin). Hypercholesterolemia was present if office notes mentioned a history of hyperlipidemia or if the patient was on an anti-hyperlipidemic medication. Heart failure was defined as the presence of a history of systolic heart failure or left ventricular ejection fraction < 50% or documented history of diastolic heart failure with EF ≥ 50%. CAD was defined as a history of previous MI/angina or history of percutaneous intervention or coronary artery bypass grafting.

### Dobutamine Stress Echocardiography Protocol

Images were obtained in the parasternal long-axis and short-axis, apical 4-chamber, 2-chamber, and apical long-axis at baseline, and after each incremental dose of dobutamine. Images were digitally stored at baseline, low, intermediate, and high doses to facilitate quad screen display and analysis. Recovery images were also obtained and stored on videotape. In the case of suboptimal digital capture quality; a tape review was performed for interpretation. Heart rate, blood pressure, and 12-lead electrocardiograms were recorded at baseline and monitored through each stage. After baseline images, dobutamine was initiated at a dose of 10 μg/kg/min and increased at 3-minute intervals to 20, 30, and up to a maximum of 40 μg/kg/min. Per our lab protocol, atropine is injected (observing standard precautions and contraindications at 0.2 mg dose increments every minute, up to a total dose of 2 mg) if ≥ 85% age-predicted maximum heart rate or an absolute heart rate of ≥ 100 had not been reached after a 3-minute infusion of dobutamine at 20 μg/kg/min). The test was terminated at the completion of the protocol or with the development of significant ischemic ST-segment shifts, intolerable symptoms, ventricular tachycardia, symptomatic hypotension (or systolic blood pressure < 90 mmHg), or severe hypertension (> 220/110 mmHg). A NDSE was defined as having a normal contractile response with dobutamine regardless of resting wall-motion abnormalities. Visual assessment of wall motion was performed using the following format: normal, mildly hypokinetic, severely hypokinetic, akinetic, and dyskinetic. The ASE standard 16-segment model, which was the recommended model by the ASE at the time of this study conception, was used for reporting of wall motion. Analysis was primarily done by visual assessment of wall motion. Ejection fraction estimation was based on visual assessment and an ejection fraction ≥ 50% was defined as normal. The decision to withhold beta-blockers and other anti-anginals was left to the discretion of the referring physician.

Electrocardiograms were designated as ischemic with the presence of ≥ 1 mm of horizontal or downsloping ST-segments 80 ms after the J-point, or if there was ≥ 1 mm ST-segment elevation in leads without significant Q-waves at baseline. Patients with a positive stress ECG but normal peak wall motion were considered to have negative DSE and were part of the study group.

### Endpoints and Definitions

Major adverse cardiac events (MACE) assessed individually and as a composite were nonfatal myocardial infarction (MI), cardiac death, and revascularization. Patients were also followed up for softer endpoints such as unstable angina. The hard MACE composite of nonfatal MI and cardiac death was also assessed. A follow-up period of 36 months was used when plotting survival curves. MI was defined by creatine kinase elevation > 2 times the upper limit of normal, or troponin elevation above the upper limits of normal for our lab assay in the setting of chest pain, or other clinical signs/symptoms suggesting cardiac ischemia. Cardiac death was documented as death related to MI (ST-elevation MI or non-ST-elevation MI), congestive heart failure, sudden cardiac death or arrhythmias. Revascularization included any percutaneous intervention or coronary artery bypass grafting. Unstable angina was defined as an accelerated pattern of chest pain with increased frequency, longer anginal duration, and decreased response to medical therapy, occurrence at rest or new onset chest pain.

### Statistical analyses

A descriptive analysis was performed comparing clinical, demographic, and echocardiography variables between AA and CA patients. Student's *t *test for continuous variables and chi square analysis for categorical variables were used. Kaplan-Meier analysis was used to create freedom-from-event survival curves for individual major clinical outcomes (non-fatal MI, cardiac death, revascularization, and any cardiac event). The log-rank test was used for comparing the survival curves between the 2 groups.

The Cox proportional hazards regression modeling was used to evaluate clinical, demographic, and stress echocardiography variables as predictors of hard MACE (cardiac death/non-fatal MI). Sub- group analysis was performed excluding those patients who failed to achieve a PMHR of ≥ 85%. The covariates included age, male gender, diabetes mellitus, hypertension, previous history of CAD/MI/revascularization, history of heart failure, hypercholesterolemia, tobacco use, chest pain, b-blocker/ca-channel blocker, LVH, EF, and PMHR ≥ 85%. From the multivariable modeling, adjusted hazard ratios with 95% confidence intervals were calculated along with p-values. All statistical analysis was performed using the SAS software (version 9.1.3). P-values less than 0.05 were considered statistically significant.

## Results

### Patient demographic and clinical characteristics

The study group with negative DSE comprised 727 patients of whom 387 were AA, and 340 CA. Mean overall follow-up was 39 ± 18 months. AA patients were younger (69.4 ± 12.6 years vs. 74.2 ± 10.7 years, *p *< .001), had higher rates of diabetes mellitus (37% vs. 29%, *p *= .01), and hypertension (91% vs. 85%, *p *= .006) at baseline compared to CA patients (Table 1). CA patients were more likely to have history of CAD (27% vs. 34%, *p *= 0.05), hypercholesterolemia (55% vs. 47%, *p *= 0.03) and prior coronary artery bypass graft surgery (11% vs. 7%, *p *= .04) compared to AA patients.

**Table 1:** Patient characteristics

	**African-American (N = 387)**	**Caucasian (N = 340)**	***p *value**
Age (years)	69.0 ± 12.6	74.2 ± 10.7	< 0.001*
Male	148/387 (38%)	151/340 (44%)	0.092
Diabetes	143/387 (37%)	97/340 (29%)	0.016*
CAD	104/387 (27%)	114/340 (34%)	0.051
History of CHF	76/387 (20%)	57/340 (17%)	0.317
Hypertension	354/387 (91%)	289/340 (85%)	0.006*
Hypercholesterolemia	183/387 (47%)	188/340 (55%)	0.031*
Tobacco use	80/387 (21%)	103/340 (30%)	0.003*
Prior MI	82/387 (21%)	76/340 (22%)	0.704
Prior PCI	39/387 (10%)	45/340 (13%)	0.184
Prior CABG	28/387 (7%)	39/340 (11%)	0.049*
Chest pain	35/387 (9%)	24/340 (7%)	0.328
B-blocker/CC-blocker	189/387 (49%)	163/340 (48%)	0.809

*Statistically significant; B = beta; CABG = coronary artery bypass grafting; CAD = coronary arterial disease; CC = calcium channel; CHF = chronic heart failure; MI = myocardial infarction; PCI = percutaneous coronary intervention

### Dobutamine stress echocardiography characteristics

AA patients were more likely to have a hypertensive response (17% vs. 4%, *p *< .001) compared to CA patients (Table 2) and less likely to achieve a target heart rate (50% vs. 61%, *p *= 0.002) despite comparable beta-blocker/calcium-channel blocker usage (49% vs. 48%, *p *= 0.8). While both groups had similar rates of preserved systolic function (ejection fraction ≥ 50%) at baseline (81% vs. 82%, *p *= 0.8), AA's had significantly higher rates of echocardiographic left ventricular hypertrophy (defined as M-mode measured septal and posterior wall end-diastolic thickness ≥ 11 mm: 70% vs. 49%, *p *< .001).

**Table 2:** Echocardiography variables

	**African American**	**Caucasian**	***P *value**
Hypotensive response	99/387 (26%)	129/337 (38%)	< 0.001*
SBP ≥ 200 and DBP ≥ 90	12/381 (3%)	2/333 (1%)	0.014*
SBP ≥ 200 or DBP ≥ 90	64/381 (17%)	13/333 (4%)	< 0.001*
LVH	271/387 (70%)	168/340 (49%)	< .001*
Ischemic ECG Change	16/385 (4%)	17/337 (5%)	0.568
Baseline WMA	86/385 (22%)	89/338 (26%)	0.211
EF ≥ 50%	314/387 (81%)	277/339 (82%)	0.843
PMHR ≤ 85%	192/386 (50%)	132/338 (39%)	0.002*

*Statistically significant; DBP = diastolic blood pressure; ECG = electrocardiogram; EF = ejection fraction; LVH = left ventricular hypertrophy; PMHR = predicted maximal heart rate; SBP = systolic blood pressure; WMA = wall motion abnormalities

### Three-year cardiac event-free survival analysis

**Figure 1:**
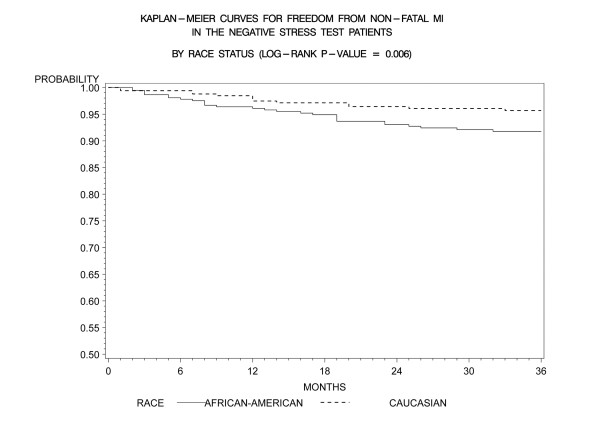
**Kaplan- Meier curves for freedom from non-fatal MI compared by patient race in negative DSE patients (log rank p-value = 0.006)**. DSE- Dobutamine stress echocardiography, MI-Myocardial Infarction.

**Figure 2:**
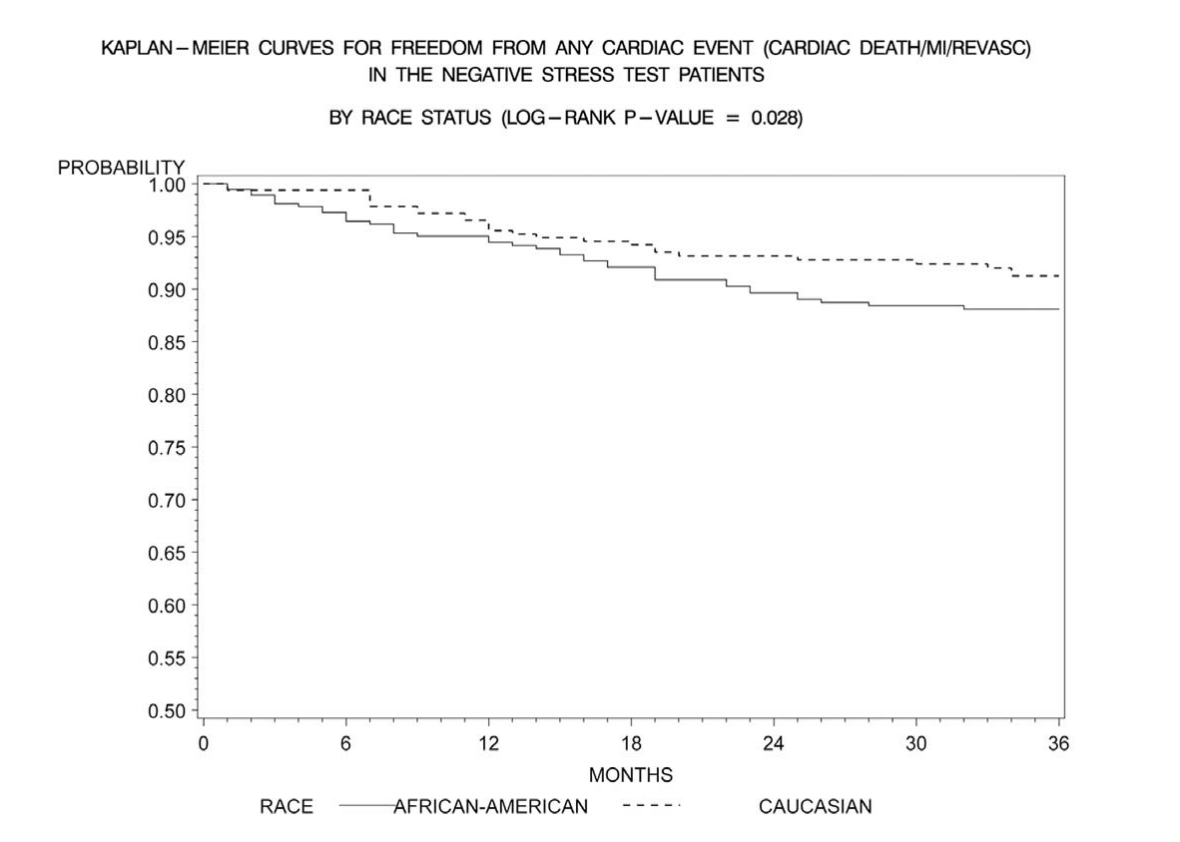
**Kaplan- Meier curves for freedom from any cardiac event (cardiac death, MI) compared by patient race in negative DSE patients (log rank p-value = 0.005)**. DSE- Dobutamine stress echocardiography, MI-Myocardial Infarction.

**Figure 3:**
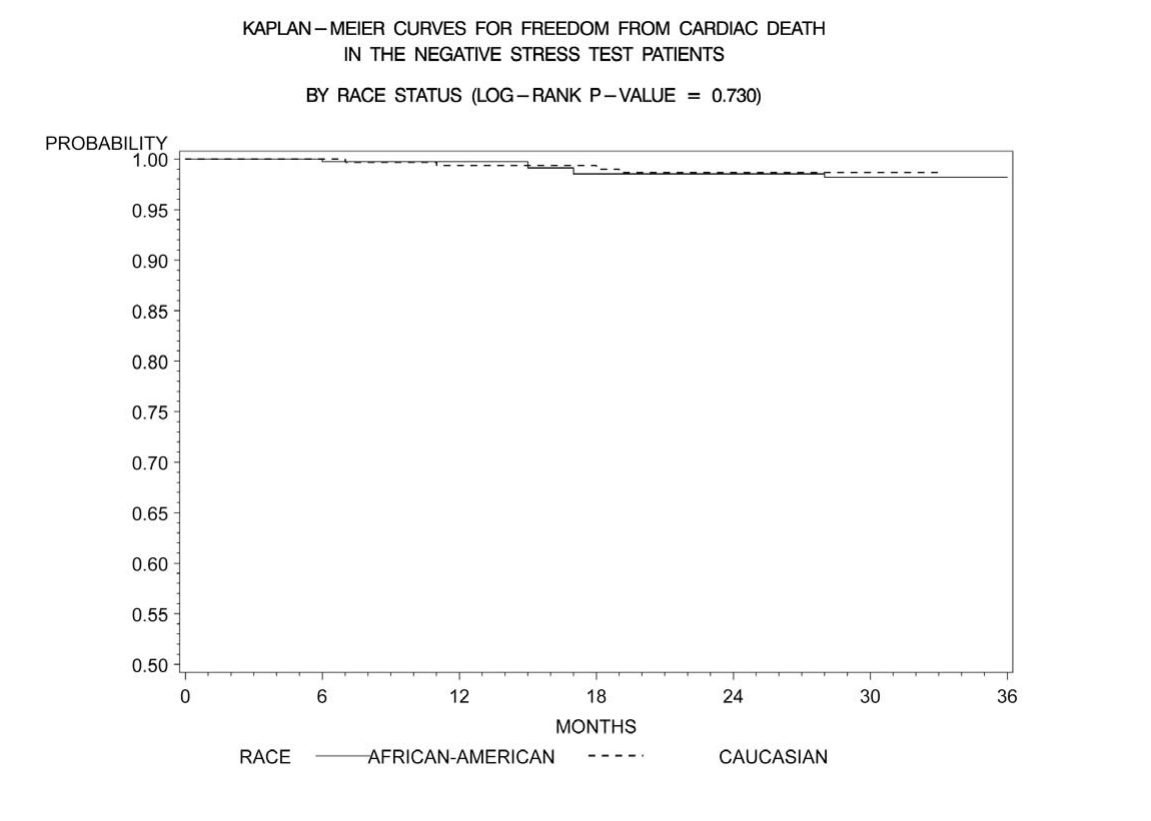
**Kaplan- Meier curves for freedom from cardiac death compared by patient race in negative DSE patients (log rank p-value = 0.730)**. DSE- Dobutamine stress echocardiography.

**Figure 4:**
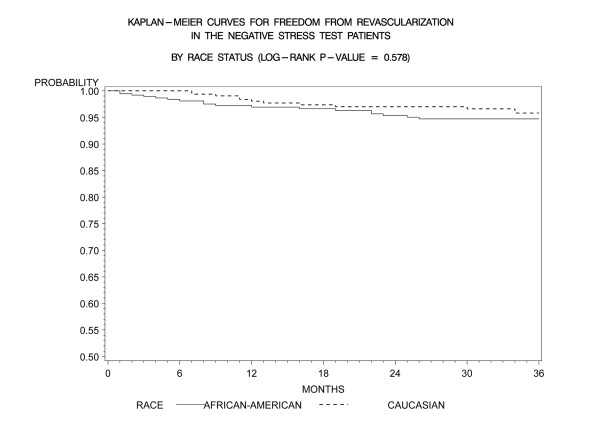
**Kaplan- Meier curves for freedom from revascularization compared by patient race in negative DSE patients (log rank p-value = 0.578)**. DSE- Dobutamine stress echocardiography.

**Figure 5:**
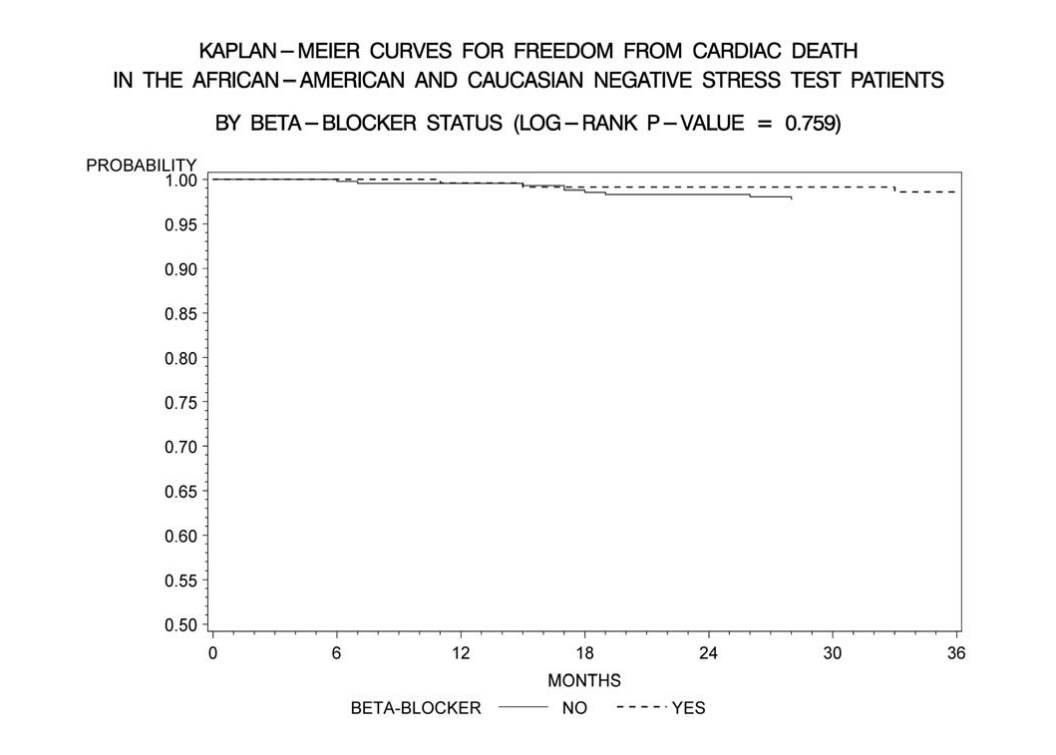
**Kaplan- Meier curves for freedom from cardiac death compared by presence of absence of beta-blocker therapy in the entire patient cohort post negative DSE (log rank p-value = 0.759)**. DSE- Dobutamine stress echocardiography.

**Figure 6:**
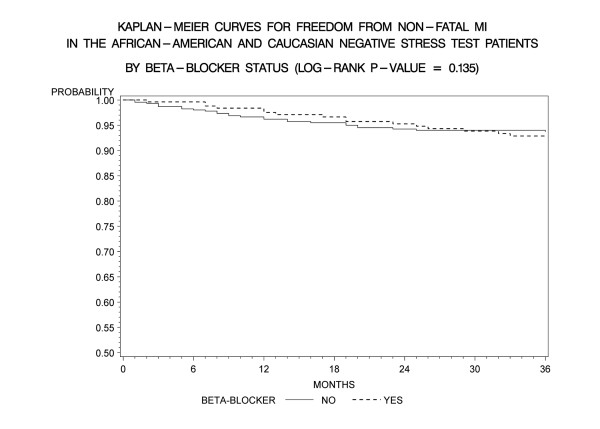
**Kaplan- Meier curves for freedom from non-fatal M.I compared by presence of absence of beta-blocker therapy in the entire patient cohort post negative DSE (log rank p-value = 0.135)**. DSE- Dobutamine stress echocardiography.

Kaplan-Meier freedom-from-events analysis showed that AA patients had a lower freedom from nonfatal MI (92% vs. 96%, *p *= 0.006), and any cardiac event (cardiac death/MI) (91% vs. 95%, *p *= .005) compared to CA patients (Figures 1 and 2). Freedom from unstable angina also trended lower in AA patients compared to CA patients (86% vs. 89%, *p *=. 08). There was no significant difference in the rates of event-free freedom from cardiac death (97% vs. 98%, *p *= .7), and revascularization (94% vs. 96%, *p *= .5) (Figures 3 and 4). At three years, rates of cardiac death were similar between both the groups (CA – 0.7% [5/727]; AA – 0.9% [7/727]), whereas rates of nonfatal MI's were higher in AA's (CA – 2% [15/727]; AA – 5% [37/727]). Patients from both ethnic cohorts were further analyzed for hard MACE (cardiac death/non-fatal M.I) based on the presence or absence of beta-blocker therapy and no differences were observed (Figures 5 and 6).

### Multivariable Cox proportional hazards regression analysis

Cox regression analysis was used to evaluate the ability of race to predict the hard MACE presence of cardiac death/nonfatal-MI after accounting for study covariates (Table 3). AA ethnicity carried a 2.83-fold higher risk for cardiac death or non-fatal MI compared to CA patients. The other significant predictor was older age (1.03-fold higher per year). Sub-group analysis performed in patients with a NDSE and PMHR ≥ 85% again demonstrated that AA race still was a significant (3.23-fold higher) predictor of cardiac death/nonfatal-MI after accounting for similar clinical and stress variables as listed above (Table 4).

**Table 3:** Risk-adjusted Cox proportional hazards regression analysis for predicting cardiac death/M.I in African-American and Caucasian patients with NDSE

	**Hazard Ratio**	**95% CL**	**P-value**
Race (African-American)	2.878	1.608 – 5.151	<0.001 *
Age in Years	1.031	1.007 – 1.056	0.011 *
Male	1.392	0.816 – 2.375	0.225
Diabetes	1.522	0.900 – 2.576	0.117
CAD/Prior MI/Prior Revascularization	1.537	0.861 – 2.746	0.146
History of CHF	1.098	0.567 – 2.127	0.781
Hypertension	1.002	0.345 – 2.910	0.997
Hypercholesterolemia	1.002	0.587 – 1.710	0.994
Tobacco Use	1.677	0.954 – 2.948	0.072
Chest Pain	1.587	0.739 – 3.407	0.236
B-Blocker/CC-Blocker	1.164	0.674 – 2.010	0.586
LVH	0.756	0.444 – 1.285	0.301
EF in Percent	0.978	0.955 – 1.002	0.071
PMHR ≥ 85%	1.344	0.788 – 2.292	0.278

*Statistically significant; CAD = coronary arterial disease; CHF = chronic heart failure; CL = confidence limits; EF = ejection fraction; LVH = left ventricular hypertrophy; MI = myocardial infarction; PMHR = predicted maximal heart rate; NDSE = Negative Dobutamine Stress Echocardiography

**Table 4:** Risk-adjusted Cox proportional hazards regression analysis for predicting cardiac death/M.I in African-American and Caucasian patients with PMHR ≥ 85% and NDSE

	**Hazard Ratio**	**95% CL**	**P-value**
Race (African-American)	3.234	1.579 – 6.622	0.001 *
Age in Years	1.049	1.012 – 1.086	0.009 *
Male	1.294	0.641 – 2.610	0.472
Diabetes	0.916	0.426 – 1.968	0.822
CAD/Prior MI/Prior Revascularization	2.607	1.205 – 5.639	0.015 *
History of CHF	1.101	0.461 – 2.633	0.828
Hypertension	1.059	0.301 – 3.729	0.929
Hypercholesterolemia	0.811	0.393 – 1.674	0.571
Tobacco Use	1.616	0.760 – 3.435	0.213
Chest Pain	2.457	0.991 – 6.088	0.052
B-Blocker/CC-Blocker	1.153	0.572 – 2.326	0.691
LVH	0.590	0.290 – 1.200	0.145
EF in Percent	0.977	0.945 – 1.011	0.182

*Statistically significant; CAD = coronary arterial disease; CHF = chronic heart failure; CL = confidence limits; EF = ejection fraction; LVH = left ventricular hypertrophy; MI = myocardial infarction; PMHR = predicted maximal heart rate; NDSE = Negative Dobutamine Stress Echocardiography

## Discussion

This is the first study to our knowledge to directly compare long-term MACE between AA and CA patients with a NDSE in an unselected patient population. Our study found that AA patients were more likely to suffer from non-fatal MI and any MACE compared to CA patients, despite a NDSE. In multivariate analysis, AA's were twice more likely to suffer from any cardiac event compared to CA patients after controlling for clinical and echocardiographic variables.

Baseline patient characteristics in our study are consistent with many prior studies showing that AA's tend to be younger, with a greater female preponderance, and have higher rates of diabetes mellitus, hypertension, and left ventricular hypertrophy, but lower rates of CAD compared to CA counterparts[22,23]. A low comparable cardiac death rate in both groups is also consistent with prior literature, demonstrating a low incidence of cardiac death in patients with a NDSE [24].

### Race and dobutamine stress echocardiography

In our study, AA patients had higher incidences of diabetes mellitus and hypertension, and concomitantly higher rates of left ventricular hypertrophy. Also, despite comparable rates of similar systolic function in both groups and lower rates of ischemic disease in AA's, these patients had worse outcomes. This is consistent with prior reports that cardiovascular mortality in AA's is more likely to result from hypertension, obesity, and diabetes mellitus, leading to increased micro-vascular disease and overall mortality rather than from CAD leading to pump failure [25,26]. Also, prior 2D echocardiography studies focusing on left ventricular hypertrophy [27] have shown that AA's are more likely to have more cardiac events despite a preserved left ventricular function [22]. While the overall incidence of cardiac death was low and similar in both groups (CA: 0.7% [5/727]; AA: 0.9% [7/727]), AA patients accounted for a significantly higher percentage of nonfatal MI's (CA: 2% [15/727]; AA: 5% [37/727]) in this study.

What could account for this? It is well known that stress echocardiographic (exercise or pharmacologic) techniques have greater specificity (with relatively lesser sensitivity) than stress nuclear techniques for epicardial CAD detection. This is at least partly related to the need for true ischemia and wall motion abnormalities for CAD detection by stress echo caused by hemodynamically significant stenosis rather than heterogeneity of flow in nuclear imaging, which may happen even in absence of significant CAD. Thus dobutamine stress echocardiography could underestimate disease in patients with mild to moderate non-obstructive CAD due to lack of ischemia and this may be confounded further by chronotropic incompetence in presence or absence of beta- blockers. Also test sensitivity can be reduced if patients are tested on anti-anginal therapy. Higher rates of nonfatal MI and unstable angina in AA patients in setting of a NDSE could imply that this group may have underlying active atherosclerosis/endothelial dysfunction, which is underappreciated by current dobutamine stress echocardiographic techniques that rely on wall-motion abnormalities to detect ischemia. Prior literature has demonstrated that AA patients have a 33% excess prevalence of hypertension and a 50% excess prevalence of diabetes [28,29], both of which are known risk factors for CAD and are associated with abnormal coronary and microvascular relaxation [30-34]. It has also been shown that when left ventricular hypertrophy and chronic hypertension are present together they seem to be the most important predictors for abnormal vasomotor reactivity to endothelium dependent/independent stimulus both in AA's and CA's, while normotensive patients (both AA and CA) show no such differences in response to coronary vasomotor stimulation [30-34]. This carries significance as shown in our study as not only is diabetes mellitus more prevalent in AA (Table 1), but also it has been shown that diabetics especially with CAD carry an increased cardiovascular risk and carry a less benign prognosis despite a NDSE [35]. As further suggested by Cortigiani et al, this high-risk cohort of patients should be evaluated by other non-invasive measures such coronary flow-reserve assessment by transthoracic stress echocardiography [36] for better risk stratification. Extrapolating on this is the interesting question of whether assessment of flow reserve abnormalities at peak dobutamine infusion in AA with NDSE could potentially identify the higher risk subgroup. This needs further study.

Based on the much higher prevalence of hypertension, diabetes, abnormal BP response and LVH, AA patients likely have a greater predilection for acquired coronary and microvascular dysfunction explaining higher non-fatal MI and any overall MACE as seen in our study. Existing echocardiographic stress testing modalities currently do not have the robustness of identifying sub clinical atherosclerosis and endothelial dysfunction. Myocardial perfusion contrast echocardiography holds this promise but is not routinely still incorporated in clinical practice. Nuclear stress imaging techniques such as SPECT relative perfusion analysis and positron emission tomography with absolute blood flow quantization could identify flow reserve abnormalities. In clinical practice dobutamine echocardiography remains robust to exclude major epicardial disease but cannot rule out non-obstructive atherosclerosis or endothelial dysfunction both of which can influence cardiac outcomes.

Despite higher rates of non-fatal MI and total MACE the entire patient group in our study had low cardiac death rates over 3 years, regardless of race. This likely reflects the characteristics of our study group where there was high prevalence of normal EF (EF being a major determinant of cardiac death). Our group has previously shown that a normal contractile response in setting of preserved EF is associated with a low cardiac death rate over a 3 year follow-up period regardless of heart rates achieved with a NDSE [37].

### Race and target heart rate achievement

It could also be argued that under recognition of CAD in our study group particularly in AA's could explain higher subsequent event rates. Only 50% of AA's compared to 61% CA patients achieved PMHR ≥ 85%, (*p *= .002), despite comparable high rates of atrioventricular nodal blocker use at the time of DSE (49% vs. 48%, *p *non significant) in our study. Nevertheless our study sub- target heart rate percentages are comparable to prior studies, which have been reported in upto 60%, in patients undergoing DSE [38-40]. Sub-group analysis of patients on and without beta-blocker therapy showed higher overall MACE despite a NDSE (87% vs. 93%, p = 0.002) but the event rate was primarily driven by higher rates of unstable angina (93% vs. 78%, p < 0.001) and re-vascularization (97% vs. 93%, p = 0.001), whereas rates of cardiac death (98% vs. 99%, p = .759) and non-fatal M.I (94% vs. 93%, p = .135) did not differ (Figures 5 and 6). These results were consistent across both ethnic patient cohorts. This likely reflects the influence of beta-blockers on potentially causing under recognition of CAD due to lack of heart rate response or reduced ischemia detection due to anti ischemic effect. Thus patients tend to present back with angina and undergo coronary angiography and revascularization.

One study specifically assessing racial differences in heart rate and blood pressure response to dobutamine, reported a 47% incidence of submaximal heart rate response despite holding beta-blockers prior to DSE [41]. That study also showed that compared to CA's, AA patients (primarily male gender) had a higher elevated baseline blood pressure and hence an increased hypertensive response, which likely lead to higher, rates of premature termination of a DSE and accounting for higher rates of submaximal tests. As shown in Tables 1 and 2, these findings are replicated in our study demonstrating higher baseline blood pressures, more LVH and higher DSE induced blood pressure responses in AA's compared to CA patients. Pharmacogenetic studies in AA's show a varied hemodynamic response to some vasodilators, leading to altered vasoreactivity [42,43]. Further pharmacological and clinical studies are warranted in this area, specifically evaluating the response to dobutamine in AA patients and whether specific beta receptor responsiveness or pharmacokinetic differences may mediate such responses.

Given the high rates of submaximal NDSE in our AA which could potentially influence cardiac event rates, we performed a sub-group analysis including only AA and CA patients who achieved a PMHR ≥ 85% from the study population. Consistent with overall results, as shown in Table 4, AA ethnicity continued to be the single most important predictor of any cardiac event carrying a 3.2-fold risk, with age (1.04-fold higher per year). and history of CAD (2.6-fold higher) being the other significant predictors.

#### Strengths and limitations

The primary strengths of this study are the large number of AA patients (387/727, 50%), the long-term follow-up (3 years) and the real world implications of patients being tested on AV nodal blocking agents. The weaknesses are those inherent in retrospective analyses, which can include documentation inaccuracies and incomplete follow-up. Furthermore AA had more co-morbidities than CA, which can drive outcomes although we attempted to account for these factors in the multivariate analysis. In addition, the results of this study cannot be extrapolated to outcomes of NDSE if patients are tested in absence of beta-blockers or calcium-channel blockers.

## Conclusion

Our study demonstrates that despite a NDSE, AA patients have a significant higher incidence of nonfatal MI and overall MACE compared to CA patients at 3 years, and AA ethnicity carries a greater than 2-fold increased risk for any cardiac event. Also, our study shows that AA and CA patients are two very heterogeneous groups, when compared both by cardiovascular risk factors and stress echocardiography variables. Interesting possibilities of fundamental racial differences in responsiveness to dobutamine is again raised in our study as in prior studies [41]. This requires further investigations focusing on pharmacogenetics of response to dobutamine in AA and CA's. Physicians should be aware that although NDSE overall is associated with low cardiac event rates, AA's have higher rates of nonfatal MI and MACE compared to CA patients. Further, due to the higher prevalence of risk factors such hypertension and diabetes mellitus in this ethnic group, these patients should be monitored closely and aggressive preventive and treatment strategies be employed to help reduce cardiovascular morbidity and mortality.

## Abbreviations

CAD: Coronary artery disease; AA: African American; CA: Caucasian; DSE: Dobutamine stress echocardiography; NDSE: Negative dobutamine stress echocardiography; ASE: American Society of Echocardiography; ECG: Electrocardiogram; MACE: Major adverse cardiac events; MI: Myocardial infarction; EF: Ejection Fraction; WMA: Wall motion abnormalities; PMHR: Predicted maximum heart rate; LVH: Left ventricular hypertrophy

## Competing interests

The authors declare that they have no competing interests.

## Authors' contributions

All authors participated in conceiving the study, design, data collection and interpretation of results. GJ performed the statistical analysis. KA coordinated the study. All authors read and approved the final manuscript.
